# *GradeAid*: a framework for automatic short answers grading in educational contexts—design, implementation and evaluation

**DOI:** 10.1007/s10115-023-01892-9

**Published:** 2023-05-19

**Authors:** Emiliano del Gobbo, Alfonso Guarino, Barbara Cafarelli, Luca Grilli

**Affiliations:** 1 Department of Economics, Management and Territory, University of Foggia, Via da Zara, 11, 71121 Foggia, FG Italy; 2 Department of Humanities, University of Foggia, Via Arpi, 176, 71121 Foggia, FG Italy

**Keywords:** Automatic short answer grading, Natural language processing, Learning analytics

## Abstract

Automatic short answer grading (ASAG), a hot field of natural language understanding, is a research area within learning analytics. ASAG solutions are conceived to offload teachers and instructors, especially those in higher education, where classes with hundreds of students are the norm and the task of grading (short)answers to open-ended questionnaires becomes tougher. Their outcomes are precious both for the very grading and for providing students with “*ad hoc*” feedback. ASAG proposals have also enabled different intelligent tutoring systems. Over the years, a variety of ASAG solutions have been proposed, still there are a series of gaps in the literature that we fill in this paper. The present work proposes *GradeAid*, a framework for ASAG. It is based on the joint analysis of lexical and semantic features of the students’ answers through state-of-the-art regressors; differently from any other previous work, *(i)* it copes with non-English datasets, *(ii)* it has undergone a robust validation and benchmarking phase, and *(iii)* it has been tested on every dataset publicly available and on a new dataset (now available for researchers). *GradeAid* obtains performance comparable to the systems presented in the literature (root-mean-squared errors down to 0.25 based on the specific tuple $\langle$dataset-question$\rangle$). We argue it represents a strong baseline for further developments in the field.

## Introduction

In the past, there has been a growing number of students enrolled at universities worldwide.^1^ Large courses have thousands of students participating, especially when using virtual classrooms. In introductory computer science and software engineering courses, classroom sizes with up to 1700 students are no longer an exception, with growth by factor five in the last ten years. The free Stanford Massive Open Online Course (MOOC) *“Intro to Artificial Intelligence,”*^2^ is started in 2011, quickly reaching 160,000 students [1]. After witnessed a period of decline, MOOCs are coming back,^3^ also due to the COVID-19 pandemic outbreak. As a result of COVID-19, higher education all over the world has moved to deliver courses online [2–5], facing issues similar to MOOCs.

Large lectures pose a problem for instructors when grading textual exercises, and the shifting of learning contexts from physical to virtual classrooms has made the evaluation of the students even more difficult. In addition, in [6] the authors highlighted the influence of favoritism and the emotional mindset on the assessment procedure. Therefore, automatic short answers grading (ASAG) systems have been introduced both in scientific research [7] and at the service of commercial solutions.^4^ Moreover, automatically scoring short student answers is important for building intelligent tutoring systems. In general, computer-aided assessment systems are particularly useful because scoring by humans can become monotonous and tedious [8]. Automatic scoring systems can help teachers save lots of time from duplication of marking student’s homework.

**Fig. 1:**
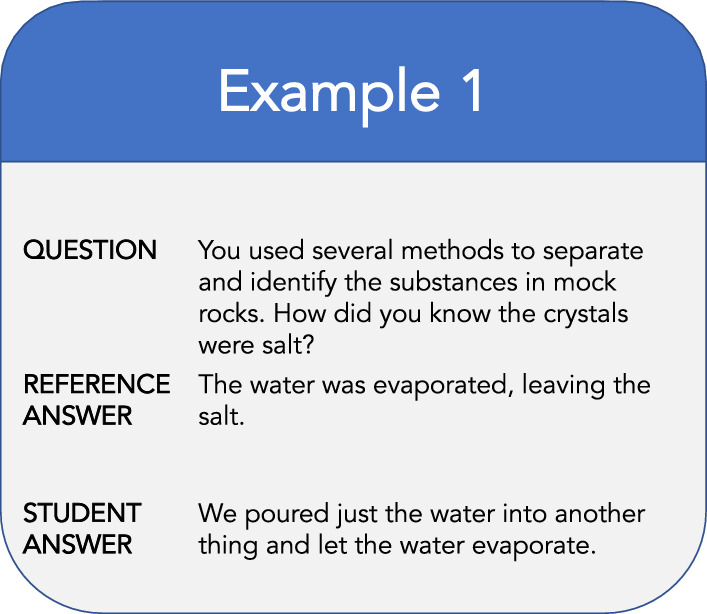
Example of question and related reference answer, and the student’s answer

One of the primary challenge in ASAG—especially with closed-ended questions—is to deal with variations in surface representations of key concepts in student answer and reference answer pairs [9] In some cases, the student answers are syntactic and lexical variations of model answers. The answer pairs may contain synonyms, polysemous words and statements that are paraphrases of each other (see Fig. 1).

The problem of finding semantic similarity of a pair of strings has been well studied in NLP literature [10]. Consequently, a variety of such similarity measures have been used—when feasible, based on the type of questions/assignments—in different ASAG systems, e.g., [11, 12]. Other systems, e.g., [13], focused on questions with no reference answers (that often appear in reading comprehension assessments) providing a grade by integrating both domain-general and domain-specific information.

*Limits*

As we will see more in detail in Sect. 2, the literature on ASAG mainly shows the following limitations: *(i)* There are a few publicly available datasets on ASAG, and they are generally very limited in size, *(ii)* works available generally did not performed experiments on all the available datasets and in most cases just on a single one, *(iii)* there are no tools or software for end-users, hence no clues on how teachers and instructors could interact with such systems and on their user satisfaction, and *(iv)* experiment’s validation conducted in previous literature is limited because authors have generally not split the datasets by question (even if in the same subject, two questions may be totally different and requiring two completely diverse—lexically and semantically—answers) or have performed just straightforward strategies such as splitting between training and testing sets. Lastly, *(v)* there is no consensus on the metrics to use, the experiments to perform and the way they should be done.

*The aim of the work*

**Fig. 2:**
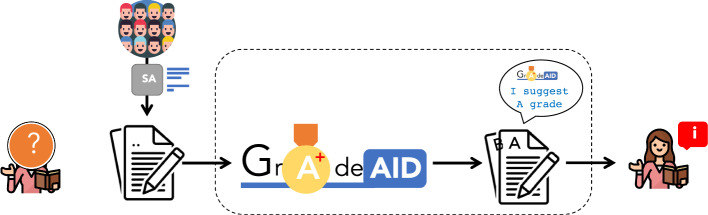
Visual abstract of our proposal. An instructor has to score the short answers (SAs) of students in a class. The answers are input to *GradeAid* that support the instructor providing a score

In this paper, we focus on developing an ASAG framework, namely *GradeAid*, to support *(i)* instructors in evaluating students questionnaires (see Fig. 2 for a sketch of our proposal), *(ii)* students’ learning by providing numerical feedback. Our attention is put toward making it usable and available to the potential stakeholders (students, instructors). On this page, we want to answer the following research questions:

- *What is the best approach for ASAG in GradeAid? In this respect, we aim to study which kind of features/text characteristics it should take into account and which machine learning method is most suitable for ASAG.*
- *How does the solution perform in different scenarios (e.g., heterogeneous datasets)?*

By answering these research questions, this paper aims to fill the gaps of the literature highlighted in the previous paragraph, and therefore offering to researchers in this field a new dataset for benchmarking, a methodological framework for experimenting and validating ASAG solutions as well as the code used so to ease other researchers in comparison with our tool. The code would serve as a “scaffold”, a basis to build and validate future ASAG systems. The impact foreseen is to ease the development of ASAG systems through offering more comparability and reproducibility to ASAG systems.

*The proposed approach*

Our idea is to exploit both lexical and semantic features of answers to provide a final grade. First, we have gathered different publicly available datasets for ASAG that are ASAP, SAG, SciEntBank, Cu-NLP which will be presented and discussed deeply in Sect. 3.1. For each dataset, we split the data into coherent subsets representing one question and the related students’ answers. The proposed approach integrates the traditional methodology of natural language processing tasks belonging to the bag-of-words approach, with the most modern technology based on state-of-the-art deep learning methodology involving the semantic of texts. Therefore, each answer is represented as the fusion between its semantic and lexical characteristics. The mixing of the two approaches has been useful (as seen in, e.g., [14] fusing lexical and embedding features) for feature augmentation and provided a richer feature matrix to be processed with machine learning approaches. Further details and motivation behind this implementation are available in Sect. 5. For the validation phase, given the size of the datasets, we employ the well-known leave-one-out cross-validation (LOO-CV). From the experiments carried out, we obtained that single regressors show better results than ensemble of them. Moreover, the best regressors where adaptive boosting, random forest and support vector regressor for all the datasets experimented, and we have obtained up to 0.25 root-mean-squared error (scores for students’ answers from 0 to 5).

Our method’s performance is comparable with (and sometimes better than) state-of-the-art ones. Furthermore, we have collected a new dataset for ASAG in Italian language and performed experiments with our method on this new dataset that proved its goodness achieving up to 0.42 root-mean-squared error. To give concreteness to our proposal, we embedded our method into *GradeAid* framework.

*Methodological contributions*

The primary contributions of our work can be summarized as follows:

- We have provided *GradeAid*, a framework for ASAG whose python-like pseudo-code is available in Appendix B, and its full code will be available at in a GitHub repository^5^ shortly;
- *GradeAid* is novel because it jointly considers lexical features and semantic features of the short answers, where the lexical features are computed with TF-IDF method, and semantic features are computed with BERT Cross-Encoder and condensed in a similarity score. Words (and the exact choice of words by students) are very important in several HE subjects, and thus, we had to include lexical analysis in our *GradeAid*. Second, the similarity between students’ answers and reference answers is crucial for the overall assessment as well as for specific subjects. *GradeAid*, in this version, is our proposal bringing together both clues and provides the solution in a not computationally demanding fashion.
- Compared to the literature, we have performed more robust experiments on ASAG with our method on all the datasets publicly available in the literature; the results show that our method’s performance is comparable with (and sometimes better than) state-of-the-art ones.
- We have introduced a new dataset for ASAG^6^ that has been used as testbed for our solution. The dataset will be published in the near future, for all researchers interested into.

*Contribution to ASAG research field*

We remark that, from the literature review, further expanded in Sect. 2, this research field lacks of standardized and well-defined approach in comparing the proposed methodologies performances: Researchers use different datasets and performance indicators to benchmark their methods and often do not share the code or pseudo-code or software to reproduce their tests. With this work, we intend to provide:

- a standardized set of datasets (based of the most commonly used in the literature), available in a public repository, and open for further contributions from other authors;
- a standard target of evaluation approach of ASAG solutions;
- a standard indicators’ set to benchmark other ASAG solutions’ performance.
- public repository for the proposed method, open to be improved by other researchers.

Therefore, the milestone of this paper is to lay the foundation for a developing more open, transparent and robust ASAG solutions.

*Organization*

The rest of the paper is structured as follows. Section 2 presents related works in the literature and a schematic comparison among such works with the one presented here. Section 3 offers an overview on methods and materials employed. Section 4 formulates and details the problem faced in this work. Section 5 presents *GradeAid* framework with its components and the methods evaluated. Section 6 shows obtained results and offers a throughout discussion about insights got. Finally, Sect. 7 concludes with final remarks and future developments.

## Related work

In this section, we report and offer details about previous literature showing contact points with the work here presented.

The issue of ASAG has been studied for a long time; indeed, different reviews and surveys concerning ASAG are available in the literature. In the following, we briefly discuss about main goal and findings of such studies in chronological order (from older to newest).

In [7], the authors surveyed ASAG systems, analyzing 80 papers published between 1996 and 2014. They mainly focused on the advancement of methods and approaches. The authors found that statistical methods were the most used to tackle automatic grading, and natural language processing techniques were widely adopted for extracting lexical, morphological, semantic and syntactic features from data. Moreover, they observed that this body of works was emerging; still there were barriers in the advancement of the research due to the impossibility of publishing the datasets employed for privacy reasons. Later, the authors in [15] pushed forward the analysis, including 44 papers published between 2003 and 2016. They studied datasets employed, machine learning (ML) techniques adopted, commonly used features and quality of results. The authors found that the most used ML method was SVM (no deep learning methods were used) and answers are represented through bag of words and word2vec mainly.

In [16], the authors reviewed works on ASAG published before 2019. Their aim was understanding how they work, mainly with regard to features employed, and what challenges in education they face and address. One of the drawbacks found was related to features extraction: Vast majority of proposals rely on feature engineering that can be time-consuming, since features need to be carefully handcrafted and selected to fit the appropriate model.

More recently, the authors in [17–19] reviewing a narrower set of articles detected an increasing use of deep learning techniques in last years with quality results which could favor the real applications.

For the sake of clarity, here we zoom into recent articles on ASAG which we will compare to. In particular, using Scopus^7^ and Web of Science^8^ databases, we have downloaded the latest articles (from 2018). The query used is available in Appendix C. After applying the method proposed by [20], and the quality filtering proposed by [21], we gathered a total of 32 articles about automatic short answers (or essays) grading (or scoring). Such articles have all employed a dataset of answers written in English language.

In the following, we first describe each work highlighting key differences with the present one, and then, we will compare on different criteria such papers.

First of all, we can distinguish between works dealing with ASAG as classification problem, and other ones that faced a regression problem, like we do here. We will take out of the deeper analysis the work by De Clercq et al. [22]. In such a paper, the authors proposed an analysis of handcrafted features for ASAG and their correlation with the final score assigned to a student’s answer. They did not develop a new method, or experimented with ML models, and hence, they had a different focus with respect to all other articles here surveyed.

### ASAG as classification problem

This section is devoted to present the works that have approached to ASAG as a classification problem.

Tay et al. [23] proposed a new method based on SKIPFLOW mechanism that models relationships between snapshots of the hidden representations of a LSTM network as it reads. Subsequently, the semantic relationships between multiple snapshots are used as additional features for scoring students answers. They obtained an average quadratic weighted Kappa (QWK)^9^ = 0.764. They compared the solution with different models, also a commercial one, namely *EASE*. It is unclear if the solution is portable in real-world scenarios with many more parameters to tune than the other works. In fact, the authors compare the parameters’ impact, observing that small variations in training time and parameters of the LSTM lead to performance degradation (lower than competitors) on scoring. The solution proposed only works in the English language.

Cai et al. [24] proposed a RNN architecture trained on a fusion between word embedding features (via GloVe) and handcrafted features (spelling mistakes, article length, etc.) for scoring students’ answers. They evaluated different RNN models and measured the correlation of handcrafted features with final scores. They obtained a QWK up to 0.80. Surprisingly, the size of deep learning models did not significantly affect the results. Deeper RNNs might overfit the small training data and yield inferior results than shallower networks. RNN with 50 hidden neurons might be powerful enough to learn latent information and features for this ASAP dataset. Furthermore, the usage of both handcrafted and GloVe features improves QWK. The solution has not been compared with others in literature and only works in English.

Chen et al. [25] proposed a novel CNN with a final ordinal regression layer for scoring. The model is trained on one-hot encoding of answers’ words (after segmentation and tokenization tasks). They obtained a QWK of 0.826. With respect to using LSTM and CNN models alone, the accuracy of the proposed solution has been greatly improved when combining CNN and Ordinal Regression. However, it is unclear if the method can work with languages other than English.

Chimingyan et al. [26] fine-tuned word2vec model for the embedding of students’ answers and added handcrafted features (grammar errors, answer length, average words length, etc.) for the automatics scoring through LSTM network. They compared LSTM with logistic regression, obtaining an accuracy of 0.32 and QWK of 0.94. They found that LSTM performs much better than logistic regression (QWK = 0.65) in scoring students answers. They found that grammar errors profoundly affect the final score as they are the most important among the handcrafted features. The method developed works only with the English language. Furthermore, as for other related works, the researchers did not compare their solution against other methods in the literature.

Wiranto et al. [27] introduced a method based on transfer learning Siamese dependency tree-LSTM network for scoring students’ answers. The model is trained with GloVe embedded answers and relevant words thereof combined with their synonyms generated through autoencoders. The experimental phase foresees different tests with and without synonyms, deleting words, adding noisy words, with transfer learning and without transfer learning. They obtained a QWK and accuracy of 0.8574 and 70.80%, respectively. Furthermore, they found that the proposed method generally has the best evaluation QWK and accuracy with transfer learning and without data augmentation. However, the validation method is not precise.

Hussein et al. [28] developed a framework that scores students’ answers and essay traits (ideas, style, organization, conventions). They explored multiple deep learning models for the automatic essay scoring task. The authors obtained a QWK of 0.851. The results show that the prediction of the traits scores enhances the efficiency of the prediction of the overall score. However, the authors foresee the use of essay traits prediction for giving feedback to students, but such a scenario has not been evaluated. Moreover, other models compared are faster than the developed ones.

In [29], the authors collect and perform an experiment on, a small dataset of answers in the Slovenian language, which are graded into three labels. Experiments encompass two-label and three-label settings. Answers are represented with word2vec, and the final grade is given through a state-of-the-art neural network model. One of the limitations of this work is that they only performed experiments with neural networks (no other methods have been compared), and they adopted training and testing split only.

In [30], the authors developed a bi-LSTM inspired by [31] with a lexical attention mechanism to score students’ answers. The model is trained with word2vec embedded answers. The attention mechanism relies on a feature capture layer that computes the weights for each word in the essay [31]. The method scored a QWK of 0.83. Their method can show critical words and sentences that lead to the final assigned/predicted score; thus, it has more interpretability than other deep learning approaches found in the literature. The model has not been evaluated against others in the literature. It does not consider the sentiment of the essay (the attitude of the students toward the essay). The model has not been tested on other datasets.

For the ASAG task, some researchers started using the state-of-the-art method for natural language processing, BERT. In Lun et al. [32], the authors proposed a method involving the usage of BERT. In particular, they fine-tuned the BERT base model to classify the students’ answers. Besides, the authors adopted data augmentation strategies: back-translation, correct answer as reference answer and swap content. The performance achieved from this model is measured using the accuracy and F1 on the SciEntBank dataset. The performance shows a peak $accuracy = 0.8277$ for the binary classification. The limitation of this method is that the score provided is just binary, not a more linear score of goodness to fit to the reference answer. Moreover, the fine-tuning of BERT model is a resource-intensive task. Similarly, in [33], the researchers used the SciEntBank dataset and BERT for automatic grading, by fine-tuning the model, in this case, with a softmax layer to provide the final score. In addition, the authors used XLNET (Extra Long Network) to compare performances. Here, the BERT approach provided better results over XLNET, with a *MeanF*1 score close to 80% for binary tasks. The authors only split the dataset one time randomly with 20% of data used for validation, therefore not performing a more robust testing with cross-validation. Also in [34], the authors proposed a method relying on the BERT model. In this case, the BERT basic model has been further trained using domain-specific knowledge of textbooks and further fine-tuned with questions-answer. The further training increased performance on test runs on proprietary and not publicly available datasets, achieving a *MeanF*1 = 83.95%. However, this approach is not directly reproducible since the dataset is not publicly available.

Lastly, an interesting approach was conceived in [35], where the authors represented the students’ answers and the reference ones as a graph. Each sentence is represented as a node, and it is connected with an edge to another type of node representing the uni-gram and bi-gram included in the very sentence. Then, the method used to grade the answer is based on a graph neural network that accounts for similarities between the reference answer’s graph and the student’s one. Furthermore, the authors provide a deep analysis of the graph features more correlated with the final grade. However, they have used only the ASAP dataset, and their experiments involve a straightforward approach of splitting the dataset into training and testing, then reserving 10% of the training set for validation purposes.

### ASAG as regression problem

This group of works is closer to the one presented here because the problem of ASAG has been approached as a regression one. Therefore, to better highlight differences and similarities, we first describe each element found in this group, and then, we sketch and summarize significant points of each of them (and ours) in Table 1. The table focuses on the following aspects:

- *Dataset*, i.e., the set of datasets employed;
- *Features*, i.e., how text answers have been represented;
- *Method*, i.e., how scores have been computed (regressors, similarity methods, and so on);
- *Validation*, i.e., whether authors have only performed training and testing (*TT*) or cross-validation (*CV*), and/or split the dataset by question (*QS*), and/or cross-validation with nested features computation—as we did here—(*CV-FC*), or, lastly, validation not clear from the paper (*NA*);
- *Code*, i.e., the availability of the code.

Hassan et al. [36] compared different embedding techniques for students’ answers, i.e., word2vec, GloVe, fasttext and Skip thoughts. The proposed model employs embedding on students’ answers and reference answers to generate vector representation of answers. Cosine similarity measure between vectors of students’ answers and reference answer is used as a feature vector to train ridge regression model for predicting students’ scores. The method achieved RMSE of 0.821. Paragraph embedding techniques resulted in being the most effective for short answers grading. The authors have not compared different regression methods for obtaining the final score. The proposal has not been evaluated on diverse datasets.

Prabhudesai et al. [14] proposed a method to score students’ answers based on *(i)* word embedding via GloVe combined with handcrafted features (e.g., number of words, length of answer, number of unique words, average word length, etc.) and *(ii)* customized LSTM that takes as input embeddings, handcrafted features and the reference/golden answer. Moreover, they compare several architectures for LSTM, like plain, deep and bidirectional (also with and without handcrafted features) to find the most suitable for the scoring problem. Lastly, authors compare their solution with those previously available in the literature. They obtained a MAE of 0.618 and a RMSE of 0.889. Their work shows how including handcrafted features improves results, especially with regard to MAE (of about 0.25). However, the approach not evaluated on other datasets (like ASAP).

Sahu et al. [37] faced the problem of scoring both as regression and classification task. They experiment a rich set of text similarity features, CBoW and TF-IDF to train machine learning regressors and classifiers whose output is then conveyed into a fusion model for the final scoring. The proposed method has also been evaluated on ASAP dataset. They achieved RMSE = 0.793 and F1 from 0.50 to 0.93. The stacked ensemble method exhibits better performance than single methods. Moreover, the alignment-based and lexical overlapping features in addition to the semantic similarity features employed in the proposed system contribute a lot toward enhancement in labeling performance as compared to previous systems in the literature. The paper does not compare further ensemble methods with the one proposed. Very short answers are not graded correctly, and in some cases, the system fails to capture sequence information of answers.

Gomaa et al. [38] propose ans2vec, a method based on skip-thought mechanism to perform students and reference answer embedding and measure their similarity. The proposed method has also been evaluated on SciEntBank dataset. They achieved a RMSE of 0.91 and a *F*1 score from 0.54 to 0.58. The result is comparable with others in the literature without using any handcrafted feature. The method is not applicable on languages other than English due to the dependency from ans2vec method.

Besesio et al. [39] proposed a series of experiments for ASAG on the ASAP datasets, but they have rescaled the scores to three integer values, i.e., 1, 2 and 3. The authors have performed experiments, using cross-validation, with word2vec, with handcrafted features and with a fine-tuned BERT model. They found that the combination of all three representations of answers (students’ and reference) minimizes the error on the regression. Differently from most related works, here the hyper-parameters of the BERT model are transparent to the research community, but the final method to provide the score is hard to get from the paper.

In [40], the authors proposed a novel fusion model which takes: *(a)* the string of the reference answer and the students answers to get the first output; *(b)* the string of the question and student answer to get the second output. Finally, the model feeds the two outputs obtained above into the a features fusion layer filtering out multiple facets of semantic features, followed by an output layer for generating the final score. The proposed method has also been evaluated on ASAP and SciEntBank datasets. The method achieved a RMSE of 0.678 and a F1 from 0.62 to 0.768. The proposed model overcome previous developed scoring systems on the datasets employed for the evaluation. The authors found that the proposed system performs poorly on long-tailed datasets. Lastly, the authors compare their solution with a narrow set of previous works, overlooking many contributions we have identified in the literature.

Tulu et al. [41] proposed an automatic scoring system based on SemSpace [42], a novel embedding approach to learn word vectors by weighting semantic relations, used to feed Manhattan LSTM network (which is precisely used for similarity tasks [43]). The proposed method has also been evaluated on CU-NLP dataset. It obtained RMSE = 0.040 on SAG dataset. The proposed method also showed its reliability and consistency with CU-NLP dataset. Authors show that their solution overcome previous ones in the literature (by at least 0.80). However, SemSpace cannot handle mis-spelled words or grammar errors.

Overall, as anticipated in the introduction of the paper, we remark that the literature on ASAG mainly shows the following limitations:

- there are a few publicly available datasets on ASAG, and they are generally very limited in size. Besides those listed in Sect. 3.1, no other work has released the dataset employed;
- works available generally did not performed experiments on all the (few) available datasets and in most cases just on a single one;
- there are no tools or software for end-users, hence no clues on how teachers and instructors could interact with such systems and on their user satisfaction;
- experiments’ settings are not uniform: Validation conducted in previous literature is limited because authors have generally not split the datasets by question or have performed just straightforward strategies such as splitting between training and testing sets.

**Table 1:** Comparison on different criteria among works identified in literature and this work

Study	Dataset	Features	Method	Validation	Code
[36]	SAG	word2vec, fasttext, GloVe	Cosine Similarity, RR	*TT*	✗
[44]	ASAP	word2vec	Similarity (WMD)	*QS*	✗
[45]	ASAP	word2vec	Similarity (WMD)	*QS*	✗
[46]	ASAP	word embedding	Similarity (WMD)	*NA*	✗
[14]	SAG	fusion of GloVe and handcrafted features	bi-LSTM	*TT*	✗
[39]	ASAP	handcrafted features, Word2Vec, BERT	MLP	*CV*	✗
[38]	SAG, SciEntBank	ans2vec [47]	Logistic Linear Classifier	*TT* $^\diamond$	✗
[48]	SAG	BoW, k-means, hamming distance	Linear regression	*TT*	✗
[49]	ASAP	GloVe	Attention NN	*CV-FC*	✗
[37]	ASAP, SAG	text-similarity based features, TF-IDF, CBoW	Stacked Regression	*CV*	✗
[32]	ASAP		BERT	*NA*	✗
[50]	ASAP	word2vec	Siamese C-BGRU	*CV-FC*	✗
[34]	Private (English)		BERT	NA	✗
[40]	SAG, ASAP	Autoencoders	MLP	*CV-FC*	✗
[41]	SAG, CU-NLP	SemSpace [42]	Manhattan LSTM	*CV-FC*	✗
This	ASAP, SAG, SciEntBank, Cu-NLP, STITA	TF-IDF, BERT Cross-Encoder	Ada, RF, SVR, Ensemble, EN, RR	*CV-FC, QS*	✓

$\diamond$ 60% training, 20% validation, 20% testing

## Materials and methods

In this section, we provide basic and background knowledge useful to better understand the subsequent sections. In particular, we detail the datasets employed (Sect. 3.1), the techniques exploited to represent students answers (Sect. 3.2), the machine learning methods experimented (Sect. 3.3) and the metrics used to evaluate the performance of *GradeAid* (Sect. 3.4).

### Dataset(s)

In this work, datasets reported in Table 2 have been exploited for building *GradeAid* framework. The table lists all datasets found by analyzing the related literature.

**Table 2:** Publicly available datasets identified in reviewed papers

Dataset	Language	# Samples	Score/grade range	Subject
ASAP$^{a}$	English	17,043	${\mathbb {N}}[0,3]$	*Mixed*: Science, English, Arts, Biology, etc.
SAG$^{b}$	English	2558	${\mathbb {R}}[0,5]$	Computer Science
SciEntBank$^{c}$	English	139	$\{0,1\}$	Science
CU-NLP$^{d}$	English	171	${\mathbb {N}}[0,100]$	Computer Science

${}^{a}$https://www.kaggle.com/c/asap-sas

${}^{b}$https://github.com/dbbrandt/short_answer_granding_capstone_project

${}^{c}$https://github.com/dbbrandt/short_answer_granding_capstone_project

${}^{d}$https://bmb.cu.edu.tr/uorhan/CuNLP.htm

Each dataset is built differently from the others. The ASAP dataset provides scores by two annotators in the range ${\mathbb {R}}[0,3]$. We scaled such scores in the range ${\mathbb {R}}[0,5]$ as other datasets in Table 2 and computed the average between the two annotators scores. We remark the dataset ASAP is composed of short text answers that the official page refers as essays. We have used them as short answers, and we had to include the reference answers. To obtain such reference answers, our strategy implies to use the highest scored ones among those provided by students.

Within the SciEntBank dataset, we only found correct/not correct labels. Therefore, to uniform such dataset with the others in Table 2, we asked two experts in the field of science and chemistry to provide grades for the students’ short answers (as similarly done in ASAP dataset). We measured their agreement through the Cohen’s kappa [51], obtaining a value of $k=0.642$, thus a substantial agreement. The scores are subsequently obtained by averaging the experts’ grades.

**Fig. 3:**
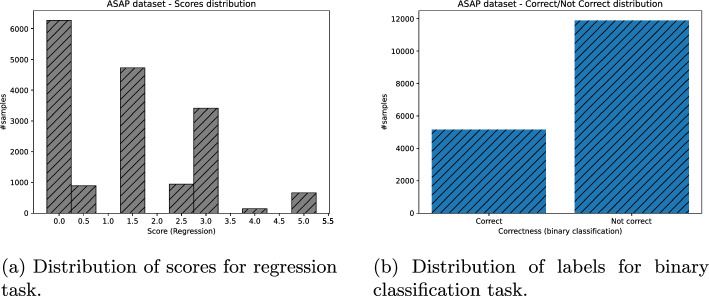
ASAP dataset distribution of scores (and their binary labels counterpart)

**Fig. 4:**
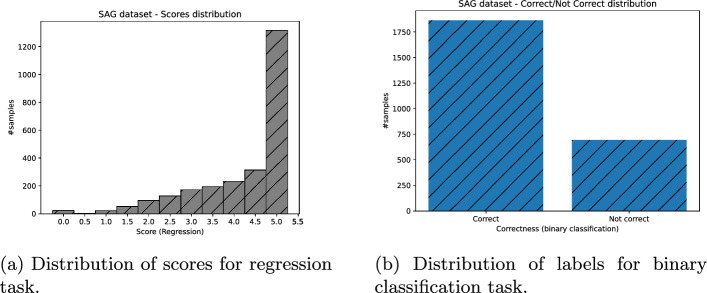
SAG dataset distribution of scores (and their binary labels counterpart)

**Fig. 5:**
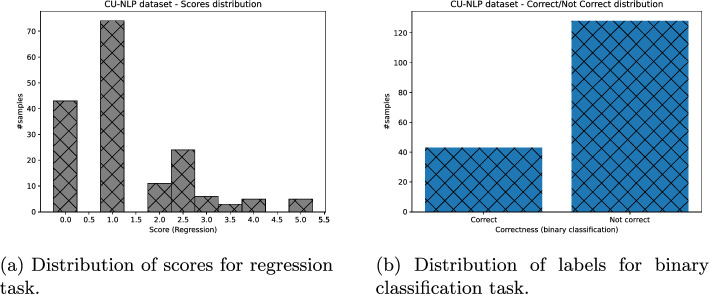
CU-NLP dataset distribution of scores (and their binary labels counterpart)

**Fig. 6:**
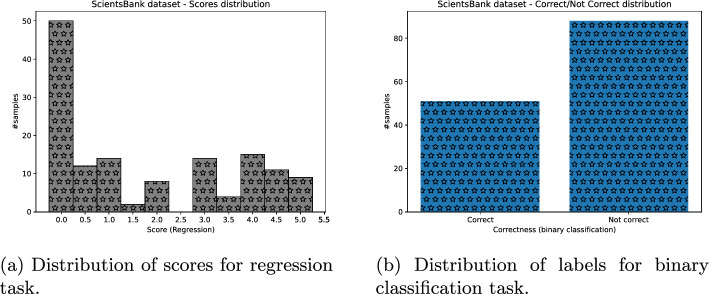
SciEntBank dataset distribution of labels (and their scores counterpart)

Lastly, we obtained the binary assessment of students’ answers, for all datasets missing it, as follows: Each answer whose score was greater or equal to 2.5 was put to “correct” class, otherwise “not correct” (Figs. 3, 4, 5, 6).

### Short answers representation

There are different ways to represent short answers: a *lexical representation*, and a *semantic representation*. For what concerns the *lexical representation*, one of the most used techniques is Term Frequency-Inverse Document Frequency (TF-IDF). This approach is a well-known approach to natural language processing (NLP) tasks that is part of techniques representing the textual data as bag of words (BoW). This name is derived to the concept of ignoring words order in documents, and focusing on the presence of the words in the specific document. Many NLP tasks showed to be managed with this simplification (e.g., email phishing detection [52], management research [53], fake news detection [54] and ASAG as well [55]). Not considering the words order, these methodologies usually have no benefit from grammar and generic words, such as “the”, “with”, “be” and “go”. The TF-IDF methodology creates a documents-terms matrix, where the documents are represented on the rows, and on the columns there is the vocabulary of all the words present in the corpus. Each cell in the matrix indicates the frequency of term in the document, multiplied for the inverse of the frequency of word across all the documents in the corpus. The first indicates is useful to manage documents of different lengths, and the second is useful to reduce the weight of words too common in the corpus, typical of stopwords.

Formally, let *k* be a term/keyword, *d* a document and *D* the set of all documents. We define $TF(k) = \frac{{\texttt {count}}(k,d)}{{\texttt {wordcount}}(d)}$, i.e., the number of times *k* appears in *d* (count(*k*, *d*)) divided by the total number of words/terms in *d* (wordcount(*d*)). We define $IDF(k)=\log _e\frac{|D|}{{\texttt {countDocs}}(D,k)}$, where countDocs(*D*, *k*) returns the number of documents containing *k*. Then, the TF-IDF is computed as follows: $\texttt {{TF-IDF}}(k,d,D) = TF(k,d) \times IDF(k,D)$. Thus, *d* is then described as an array whose length is equal to all the keywords/terms in *D*, i.e., $A_d=[{\texttt {TF-IDF}}(k_1),\ldots , {\texttt {TF-IDF}}(k_N)]$, where *N* is the total number of terms in *D*.

Concerning the *semantic representation*, in the NLP field there have been introduced several techniques to understand the meaning of words or sentences for purposes ranging from question answering [56] to opinion mining [57], cybersecurity studies [58] and law [59]. In the last years, word embedding has established itself as one of the most popular representation methods of document vocabulary [60]. Among its capabilities we can cite that of capturing the context of a word in a document, semantic and syntactic similarity, relation with other words. Word2vec [61, 62] is the most popular technique in this field. It uses the conditional probability *P*(*w*, *c*) to predict the target word *w* based on its context *c*. It has been used for a variety of tasks, e.g., finance-relating text mining [63], question answering [64], as well as ASAG systems [26, 44, 45, 50].

The success of neural networks-based methods for computing word embeddings has motivated the proposal of several methods for generating semantic embeddings of longer pieces of text, such as sentences, phrases or short paragraphs [65]. They are methods to embed a full sentence into a n-dimensional vector space. These sentence embeddings retain some properties, as they inherit features from their underlying word embeddings (e.g., semantic similarity).

The current state of the art for NLP tasks relies on the usage of BERT (Bidirectional Encoder Representations from Transformers) [66] and its related extensions, such as roBERTa [67]. BERT is designed to pre-train deep bidirectional representations from an unlabeled text by jointly conditioning on both left and right context in all layers [66]. BERT is a two-step framework. The first step is pre-training; during this step, the model is trained on unlabeled data over different pre-training tasks. The second step is fine-tuning; the BERT model is first initialized with the pre-trained parameters, and all of the parameters are fine-tuned using labeled data from the downstream task [66]. The pre-training phase works by jointly training the transformer network on two tasks: (i) predicting the masked words in the sentence (masked model language) and (ii) predicting the next sentence (next sentence prediction). Further details of BERT implementation are disserted in [66]. Once the pre-trained BERT model is achieved, it can be fine-tuned with additional output layers to create models for a wide range of tasks, such as question answering and language inference, without substantial task-specific architecture modifications [66]. For this research, we involved BERT cross-encoder: In this approach, two sentences are provided to the model, producing an output value between 0 and 1, indicating the semantic similarity between sentences.

### Machine learning methods for ASAG

One of the main techniques used in ML consists in training a model through a supervised learning algorithm which analyzes training data (observed samples) and produces an inferred function which can be used for mapping new samples. ASAG systems in the literature—see Sect. 2—face the problem of giving a score to students answers in two different ways, i.e., regression task (approximating a mapping function from input variables to *continuous* output variables) or classification task (approximating a mapping function from input variables to *discrete* output variables). In our approach, *GradeAid*—through supervised learning—exploits regressors (mapping function) to map the model/reference answers and the students ones (input) in a suitable score for each specific students answer.

Several regression methods have been proposed in the literature. To build our system, we have considered the following: support vector regressor (SVR), random forest regressor (RF), ElasticNet (EN), ridge regression (RR) and adaptive boosting regressor (Ada). The choice of such methods is justified by their widespread usage in several studies and applications. All the methods are available in *scikit-learn* Python library.^10^

### Metrics for ASAG performance assessment

In this work, we adopt standard metrics used by previous ASAG systems, and, as we will see (Sect. 4), we model the ASAG problem as regression task. In the following, we report the metrics employed to measure the performance of *GradeAid*.

*Regression*. Let $y_i$ be the target value (known score), $\hat{y_i}$ be the model’s predicted value (predicted score), and *N* be the samples, to evaluate *GradeAid* in regression task, we use the following metrics:

- *Mean Squared Error* (MSE): $MSE=\frac{1}{N} \sum \nolimits _{i=1}^N (y_i - \hat{y_i})^2$ It measures the square of differences between predictions and target values and computes the mean of them.
- *Root-Mean-Squared Error* (RMSE): $RMSE = \sqrt{MSE}$ The use of RMSE is very common, and it is considered an excellent general-purpose error metric for numerical predictions.
- *Mean Absolute Error* (MAE): $MAE=\frac{1}{N} \sum \nolimits _{i=1}^N \vert y_i - \hat{y_i}\vert$ It is known as a *scale-dependent accuracy* and therefore cannot be used to make comparisons between series using different scales.
- *Normalized Root-Mean-Squared Error* (NRMSE): $NRMSE = \frac{RMSE}{{\overline{y}}}$ While RMSE provides an absolute error metric, this measure is strictly related to the absolute scale of the distribution. Therefore, it lacks the capability to directly show the performance of the proposed method on different dataset. The normalized version of this indicator scales the error based on the true mean of distribution. This index allows us to compare the performance of the method on different datasets and so the error made varying the dataset. MAE and MSE are dependent on the datasets where the regression is made.

## Problem description

ASAG problem has been modeled as a supervised machine learning problem. Let $Q=\{q_1,q_2,\ldots ,q_m\}$ be the set of questions posed to students. Let $S=\{s_1,s_2,\ldots ,s_n\}$ be the set of students answers to the different questions in *Q*. $A = \{a_1,a_2,\ldots , a_k\}$ represents the set of *k* responses such that a unique one-to-one mapping exists between *Q*, *A* and *S*. Lastly, let $R=\{r_1,r_2,\ldots ,r_m\}$ be the set of reference/model answers to the questions such that $r_i$ is the reference answer to $q_i$. Furthermore, we define a set of grades $G=\{g_1,g_2,\ldots ,g_h\}$ that are assigned to students answers. Thus, the ASAG problem is formulated with the following input and output specifications:

- **Input**: A pair of short answers $(r_i, a_j)$ representing reference answer $r_i$ to a question $q_i$ and student answer $a_j$ to $q_i$;
- **Output**: Assignment of score *s* to student answer (regression task) based on the extent of similarity between the student answer and the respective reference answer.

The problem of ASAG (regression task) can be mathematically formulated as follows:

- the ASAG regression model is trained with a data set of $<a_j, r_i, g_j>$ tuple. Therefore, we want to learn a regression model $reg:(a_j,r_i)\rightarrow s\in {\mathbb {R}}$, i.e., $s=w\cdot X$ where *w* refers to the regression coefficients, and *X* refers to the input feature vector obtained by processing $(a_j,r_i)$.
- The scoring performance of the regression model is measured using the following evaluation metrics: RMSE, MSE, MAE and NRMSE (see Sect. 3.4).

## *GradeAid*: our framework

**Fig. 7:**
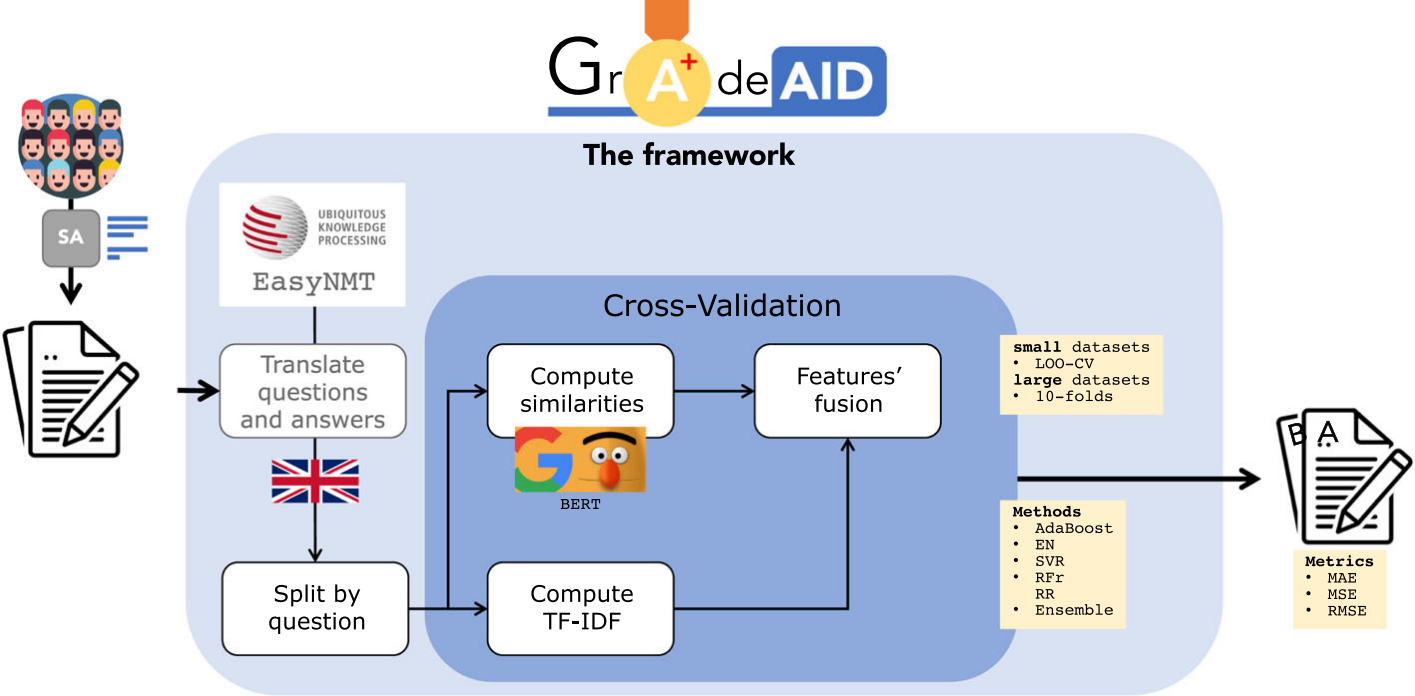
*GradeAid*: the framework. It takes students’ answers and the reference answer (it translates the texts in English if needed), and it splits the dataset provided based on the questions. During a cross-validation, at the same time, it computes similarity between students’ answers and reference answer, and TF-IDF matrix. Finally, the lexical features (TF-IDF) and semantic features (similarity) are concatenated for the prediction of the score

The framework proposed in this research aims to provide numerical feedback to students for learning purposes. A graphical representation of the framework is available in Fig. 7. The main challenge for a real application of this methodology is usually related to the datasets’ size. There a no large training datasets available to properly train the model. Moreover, the framework, namely *GradeAid*, is meant to be applicable to different languages, minimizing the need of specific tuning.

*GradeAid* works creating a model specific for each open-ended question subject to evaluation. To proceed in the creation the model, the input is the training dataset of answers labeled with a score between 0 and 5, and the reference answer (that can be or the answer provided by the teacher or, if not available, the answer with the highest score).

For answers provided in language different from English, the first block of the framework implies the usage of the EasyNMT package, a state-of-the-art machine translator for more than 100 languages.^11^ It is used to translate the corpus to English language.

**Fig. 8:**
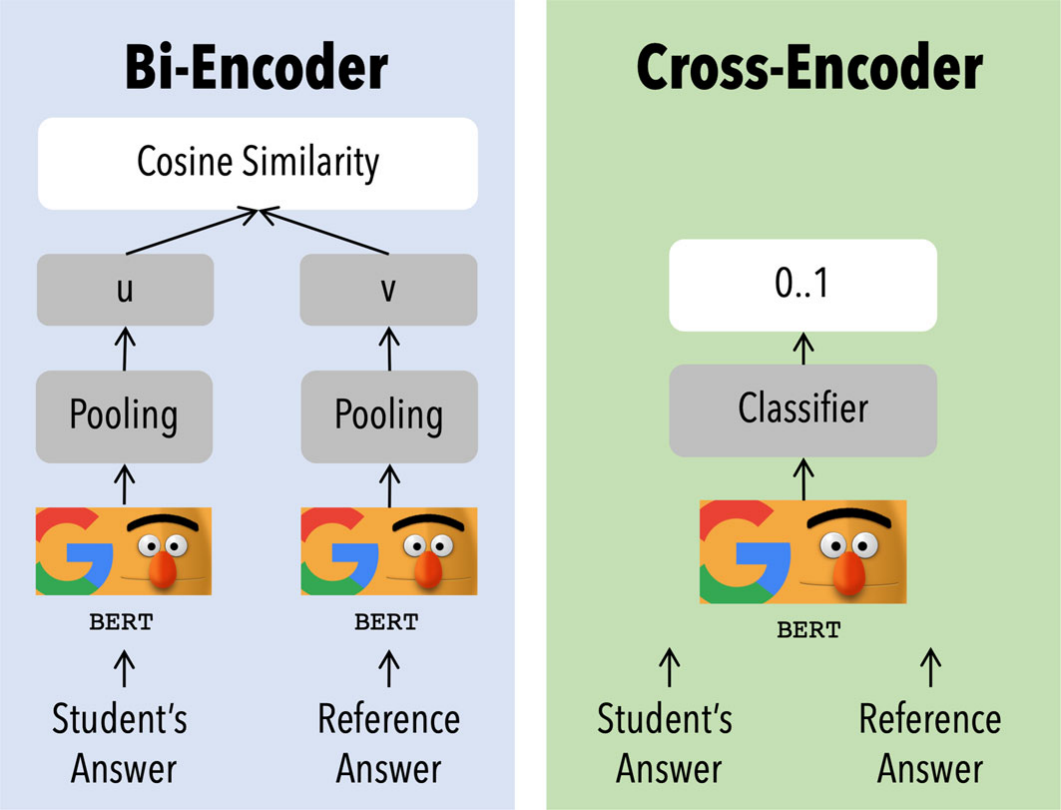
SBERT model used in this paper (left) vs BERT model as extractor through sentence embedding (right). Adapted from https://www.sbert.net/examples/applications/cross-encoder/README.html

The next block involves the construction the feature matrix from the corpus. This step consists of two different parallel operations: The well-established feature extraction method, TF-IDF, is applied to the dataset to build the matrix of words occurring in the documents; in parallel, a BERT Cross-Encoder with the pre-trained “cross-encoder/stsb-roberta-large” model is run, to build a the similarity scores between each student’s answer and the reference answer. BERT Cross-Encoder model, opposed to BoW methodologies, is trained to provide as output the semantic similarity score between a document (text) with another one. This implementation differs from bi-encoders implementations, where the sentences are passed separately to BERT to compute the embeddings (therefore used as extractor), with a final function that computes similarity. In this case, the sentences are passed to the model simultaneously that directly provides the similarity as output. This is more clear in Fig. 8. The similarity is expressed with a value between $-1$ (opposite meaning) and 1 (same meaning). The details of the implementation of this BERT configuration are further explored by [67].

The usage of these two methodologies for the features matrix construction relies on two relevant consideration about the evaluation of questionnaires: *(i)* The usage of specific words, coherent with the question, is usually a good indicator of the answer’s quality, and *(ii)* it is also relevant for the final evaluation and scoring that the sentences/answers of students are semantically correct and coherent with the reference answer.

The concatenation of the TF-IDF matrix with the similarity scores is the final feature matrix to be processed by regression methods. For the TF-IDF processing, the corpus is prepared with typical NLP preprocessing steps, lowercasing, stopwords removal (stopwords list got from the R Text Mining Solution project^12^) and not-alphabetic words removal. Instead, the processing with BERT Cross-Encoder is performed directly on the raw corpus. The feature matrix generated can be processed from every machine learning method. In the experiments, we compared several regression methods which can be found in Sect. 3.3. The final output is a score of the answer.

### Experiment

**Fig. 9:**
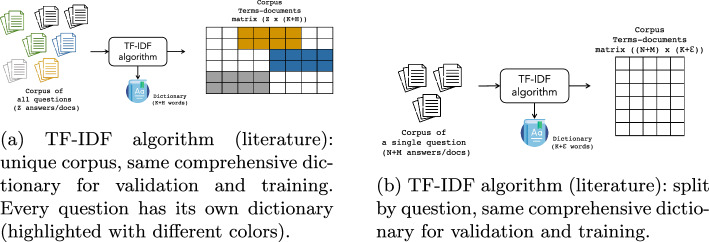
How TF-IDF algorithm can be applied in ASAG problems

**Fig. 10:**
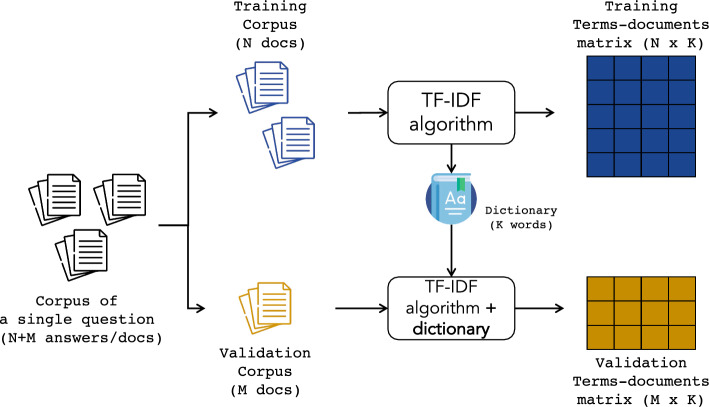
TF-IDF algorithm (this paper): split by question, dictionary resulting from training is used for applying the algorithm on the validation

Our experiments’ setting revolves around four significant points: *(1)* the split by question, *(2)* TF-IDF strategy used, *(3)* the cross-validation approach and *(4)* the combination thereof:

1. Implementing an experimental strategy for this task presents some difficulties: Often, datasets have a small size, while in some cases, they are large. Thus, finding a strategy to make results effectively comparable is not straightforward. Moreover, the small size of datasets limits rigorous benchmarking. Considering the aim of the ASAG and specifically *GradeAid*, i.e., providing feedback to students for learning, we decided to implement a strategy to simulate the most realistic use case. We treat each question (and therefore students’ and model answers) in datasets as a separate dataset, even if some questions share the same domain/context. While some approaches could benefit from other questions fusion, our implementation would not benefit from it and, on the contrary, would be misled by the mixture.
2. TF-IDF Algorithm builds the matrix and defines the number of columns in the matrix based on the vocabulary size extracted from the training dataset. Usually, each question has a specific typical dictionary for answers. Therefore, mixing answers would lead to the creation of a TF-IDF Matrix largely sparse (Fig. 9a). Instead, applying TF-IDF to a single question creates less sparse feature matrices (Fig. 10).
3. For what concerns the validation, cross-validation is the primary approach we used to measure the performance of our methods. For large datasets, such as ASAP, we performed tenfold cross-validation, while for smaller datasets, SAG, SciEntBank and Cu-NLP we used the leave-one-out cross-validation (LOO-CV). In the former case, for each question, the answers are split into ten blocks (folds), and in ten iterations, nine blocks are used for training the model, and one is used to validate results. Finally, in the last case, the reduced size of the dataset suggested us to run as many iterations as the number of samples in the dataset, leaving one sample out for validation of the model in each iteration.
4. Lastly, another significant point of our experiment methodology is as follows. In many cases of NLP, TF-IDF is performed before cross-validation. We decided to apply TF-IDF after the division between training and validation sets. This helps to simulate the actual use case, where the model is used to predict (totally) new answers of new students, with new words that have not been accounted for from the training TF-IDF. In this regard, Fig. 10 helps to describe the approach followed: Considering a dataset of $N+M$ answers, this is split into two parts, i.e., the training with *N* documents and validation with *M* documents. The TF-IDF algorithm is applied to the training dataset, generating the feature matrix based on the dictionary of *K* words used in training. The dictionary and the IDF information are retained and used as a base to compute the feature matrix for the validation corpus, which will have the same number of column of the training (*K*), and the term frequencies are weighted with the IDF weight of the training dataset.

We ran 50 times the whole experiment for each dataset and for each parameters combination so to achieve more robust results.

### Results

Tables 3, 5, 7, 9, and 13 show the results of the proposed framework, over all datasets described in Sect. 3.1. The tables report the MAE, RMSE and NRMSE achieved by all the regressors in Sect. 3.3 scoring every short answer (split by question) in each dataset. For MSE, please refer to Appendix D.

In the tables, lower values indicate better fit of the method. MAE, MSE and RMSE, however, are not absolute index of good fit, because their maximum value is dependent from the specific dataset, while NRMSE is scale independent and can be compared across datasets (see Sect. 3.4 for more details).

To increase the interpretability of the goodness of results, in the tables we provide, for each question, also the respective value of MAE, RMSE and NRMSE, for the middle regressor (*Middle reg.*), average regressor (*Avg. reg.*), and worst regressor (*Worst reg.*). These can be considered as “dummy regressors” and are useful as baseline to compare the performance of our methods. *Worst reg.* predicts always the worst value, i.e., the output for each answer is the further score possible from the true value. Therefore, its performance represents the worst that can be achieved from a regressor. Instead, *Avg. reg.* predicts always the average (mean) value of the answers for that questions. In this case, the value of MAE, MSE and RMSE of this regressor represents, respectively, the concept of average absolute deviation, variance and standard deviation. Lastly, *Middle reg.* always predicts the score in middle the minimum and maximum possible: In our case, the scores go from 0 to 5, and therefore, the middle score is 2.5.

The most evident insight is that the proposed method, with every regressor, always outperforms the dummy regressors. Considering the singular regressor methods, the best performing are SVR, RF and Ada. Poorer results are achieved from the ensemble of the selected methods. The goodness of results varies according to the dataset and questions. The best results are achieved on the ASAP dataset.

Tables 4, 6,  10, 14, and 8 show the comparison of performances of the proposed methods of this research with *Avg. reg.*, since this dummy regressor was the best among such. This comparison allows us to directly compare the performance of the regressors. Values reported are given from the ratio between the regressor RMSE and the average regressor RMSE. The formula is

$$\begin{aligned} Performance\,Ratio = 1 - \frac{NRMSE_{regressor}}{NRMSE_{Avg.Reg.}}. \end{aligned}$$

When *PerformanceRatio* is equal to 1.00, it means a full improvement of the regressor (i.e., the regressor has a $RMSE = 0$). Instead, when equal to 0.00 it indicates an “improvement” comparable to average regressor ($NRMSE_{regressor} = NRMSE_{Avg.Reg.}$). Lastly, values lower than 0.00 indicate the worsening of the regressor’s performance.

The best *PerformanceRatio* (the peak) is 0.59 for ASAP, and the two best performing regressors are SVR and RF. For Cu-NLP, RF performed very well and Ada outperformed the other regressors with a peak of 0.38. In SAG, RF outperformed the other regressors, with a peak of 0.47. It is very interesting to observe that the SVR regressor, in this case, performed worse than the baseline. Concerning the SciEntBank dataset, best performance has been achieved by RF as well, with a peak of 0.25.

In summary, the proposed methods are always able to perform ASAG with good results. However, these are not stable, and although there are two regressors with generally exciting results (SVR and RF), the performance tends to oscillate between questions and datasets. We expected ensemble could provide better results compared to single learners, but the table shows how some single learners have more potentials.

**Table 3:** Results obtained on ASAP dataset with tenfold CV

Method	ASAP														
	*q1*			*q2*			*q3*			*q4*			*q5*		
	MAE	NRMSE	RMSE	MAE	NRMSE	RMSE	MAE	NRMSE	RMSE	MAE	NRMSE	RMSE	MAE	NRMSE	RMSE
Ada	1.02	0.50	1.23	1.04	0.57	1.29	0.62	0.48	0.76	0.68	0.69	0.80	0.47	1.39	0.67
EN	1.08	0.52	1.28	1.12	0.45	1.30	0.76	0.54	0.86	0.80	0.78	0.90	0.59	1.79	0.86
SVR	0.85	**0**.**42**	**1**.**06**	**0**.**97**	**0**.**40**	**1**.**17**	**0**.**56**	**0**.**44**	**0**.**70**	**0**.**55**	**0**.**58**	**0**.**67**	0.39	1.35	0.65
RF	**0**.**84**	0.44	1.09	0.98	0.42	1.21	0.56	0.45	0.72	0.55	0.59	0.69	**0**.**36**	**1**.**30**	**0**.**63**
RR	1.06	0.52	1.28	1.11	0.45	1.29	0.76	0.54	0.86	0.79	0.77	0.89	0.60	1.77	0.85
Ensemble	1.00	0.48	1.20	1.01	0.42	1.22	0.62	0.47	0.76	0.70	0.72	0.84	0.50	1.58	0.76
*Middle reg.*	1.52	0.70	1.73	1.42	0.57	1.65	1.13	0.85	1.36	1.43	1.41	1.64	2.15	4.68	2.25
*Worst reg.*	4.02	1.65	4.10	3.92	1.39	4.01	3.63	2.31	3.70	3.93	3.46	4.01	4.65	9.79	4.70
*Avg. reg.*	1.63	0.79	1.97	1.75	0.72	2.09	0.80	0.64	1.02	0.79	0.88	1.02	1.32	3.04	1.46

	*q6*			*q7*			*q8*			*q9*			*q10*		
Ada	0.77	2.11	0.89	1.04	0.97	1.15	0.99	0.61	1.15	0.73	0.47	0.86	0.65	0.45	0.87
EN	0.80	2.23	0.94	1.15	1.01	1.21	1.09	0.65	1.22	0.81	0.49	0.90	0.71	0.46	0.89
SVR	0.42	1.59	0.67	0.84	**0**.**88**	**1**.**05**	** 0.87**	**0**.**57**	**1**.**07**	0.60	**0**.**41**	**0**.**76**	**0**.**51**	**0**.**38**	**0**.**75**
RF	**0**.**27**	**1**.**52**	**0**.**64**	**0**.**82**	**0**.**88**	**1**.**05**	0.88	0.58	1.09	**0**.**58**	**0**.**41**	**0**.**76**	0.55	0.40	0.78
RR	0.79	2.26	0.95	1.16	1.02	1.22	1.07	0.64	1.21	0.83	0.49	0.91	0.71	0.46	0.89
Ensemble	0.47	1.93	0.81	0.99	0.92	1.09	0.97	0.60	1.12	0.71	0.46	0.85	0.60	0.44	0.85
*Middle reg.*	2.28	5.59	2.35	1.68	1.58	1.89	1.26	0.80	1.50	1.15	0.75	1.38	1.08	0.65	1.27
*Worst reg.*	4.78	11.45	4.81	4.18	3.58	4.27	3.75	2.05	3.85	3.64	2.03	3.73	3.58	1.87	3.64
*Avg. reg.*	1.47	3.73	1.57	1.25	1.18	1.40	1.25	1.05	1.98	1.41	0.68	1.25	0.95	0.62	1.21

*Middle reg.* = it always predicts 2.5, i.e., the middle score. *Worst reg.* = it always predicts the worst value (e.g., score is 1, it predicts 5, score is 3, and it predicts 0). *Avg. reg.* = it always predicts the average score among the outputs

Bold values indicates the best results

**Table 4:** Comparison of methods’ performance with respect to *Avg. Reg.* (it always predicts the average value among the outputs) on ASAP dataset

Method	ASAP									
	Improvement with respect to *Avg. reg.*									
	*q1*	*q2*	*q3*	*q4*	*q5*	*q6*	*q7*	*q8*	*q9*	*q10*
Ada	0.37	0.21	0.25	0.22	0.54	0.43	0.18	0.42	0.31	0.27
EN	0.34	0.38	0.16	0.11	0.41	0.40	0.14	0.38	0.28	0.26
SVR	**0**.**47**	**0**.**44**	**0**.**31**	**0**.**34**	0.56	0.57	**0**.**25**	**0**.**46**	**0**.**40**	**0**.**39**
RF	0.44	0.42	0.30	0.33	**0**.**57**	**0**.**59**	**0**.**25**	0.45	**0**.**40**	0.35
RR	0.34	0.38	0.16	0.13	0.42	0.39	0.14	0.39	0.28	0.26
Ensemble	0.39	0.42	0.27	0.18	0.48	0.48	0.22	0.43	0.32	0.29

Positive numbers indicate an improvement in performance, and conversely, negative numbers indicate a worsening

Bold values indicates the best results

**Table 5:** Results obtained on Cu-NLP dataset with LOO-CV

Method	Cu-NLP					
	*q1*			*q2*		
	MAE	NRMSE	RMSE	MAE	NRMSE	RMSE
Ada	0.81	0.70	1.00	**0**.**64**	**0**.**59**	**0**.**81**
EN	**0**.**75**	0.77	1.09	1.43	1.37	1.90
SVR	0.76	0.77	1.09	1.30	1.25	1.73
RF	0.76	**0**.**67**	**0**.**94**	0.64	0.60	0.84
RR	0.80	0.74	1.05	1.31	1.25	1.73
Ensemble	0.78	0.71	1.01	0.99	1.04	1.44
*Middle reg.*	1.27	1.09	1.55	1.60	1.26	1.74
*Worst reg.*	3.74	2.73	3.85	4.09	3.00	4.15
*Avg. reg.*	0.85	0.78	1.10	1.02	0.96	1.33

*Middle reg.* = it always predicts 2.5, i.e., the middle score. *Worst reg.* = it always predicts the worst value (e.g., score is 1, it predicts 5, score is 3, and it predicts 0). *Avg. reg.* = it always predicts the average score among the outputs

Bold values indicates the best results

**Table 6:** Comparison of methods’ performance with respect to *Avg. Reg.* (it always predicts the average value among the outputs) on Cu-NLP dataset

Method	Cu-NLP	
	Improvement with respect to *Avg. reg.*	
	*q1*	*q2*
Ada	0.10	0.39
EN	0.01	$-$0.43
SVR	0.01	$-$0.30
RF	0.14	0.38
RR	0.05	$-$0.30
Ensemble	0.09	$-$0.08

Positive numbers indicate an improvement in performance, and conversely, negative numbers indicate a worsening

**Table 7:** Results obtained on SAG dataset with LOO-CV. We report here only the worst, best and the average results obtained on the questions due to the size of the dataset (83 questions)

Method	SAG								
	*q-worst*			*q-avg*			*q-best*		
	MAE	NRMSE	RMSE	MAE	NRMSE	RMSE	MAE	NRMSE	RMSE
Ada	**0**.**63**	0.52	1.54	0.46	0.19	0.82	0.19	0.10	0.31
EN	1.20	2.40	1.54	0.66	0.74	0.86	0.22	0.13	0.45
SVR	1.09	**0**.**42**	**1**.**25**	0.51	**0**.**17**	**0**.**73**	0.10	0.19	0.31
RF	1.15	0.50	1.47	**0**.**50**	0.19	0.82	**0**.**05**	**0**.**08**	**0**.**28**
RR	1.18	0.51	1.52	0.68	0.21	0.87	0.21	0.14	0.47
Ensemble	1.10	0.51	1.50	0.59	0.20	0.83	0.19	0.11	0.36
*Middle reg.*	2.41	0.83	2.46	1.87	0.48	2.02	1.00	0.36	1.20
*Worst reg.*	4.93	1.67	4.94	4.36	1.05	4.43	3.48	1.08	3.55
*Avg. reg.*	1.65	0.73	2.16	0.90	0.25	1.06	0.41	0.15	0.51

For all the results, see Fig. 14. *Middle reg.* = it always predicts 2.5, i.e., the middle score. *Worst reg.* = it always predicts the worst value (e.g., score is 1, it predicts 5, score is 3, and it predicts 0). *Avg. reg.* = it always predicts the average score among the outputs

Bold values indicates the best results

**Table 8:** Comparison of methods’ performance with respect to *Avg. Reg.* (it always predicts the average value among the outputs) on SAG dataset

Method	SAG		
	Improvement with respect to *Avg. reg.*		
	*q1*	*q2*	*q3*
Ada	0.29	0.24	0.33
EN	$-$2.29	$-$1.96	0.13
SVR	0.42	0.32	$-$0.27
RF	0.32	0.24	0.47
RR	0.30	0.16	0.07
Ensemble	0.30	0.20	0.27

Positive numbers indicate an improvement in performance, and conversely, negative numbers indicate a worsening

**Table 9:** Results obtained on SciEntBank dataset with LOO-CV

Method	SciEntBank											
	*q1*			*q2*			*q3*			*q4*		
	MAE	NRMSE	RMSE	MAE	NRMSE	RMSE	MAE	NRMSE	RMSE	MAE	NRMSE	RMSE
Ada	0.87	0.75	1.26	**1**.**05**	**0**.**72**	**1**.**50**	1.07	1.26	1.44	**1**.**12**	0.68	1.54
EN	1.51	0.98	1.65	1.81	0.94	1.96	1.15	1.23	1.40	1.48	**0.65**	**1**.**47**
SVR	1.16	0.81	1.36	2.33	1.33	2.77	1.10	**1**.**20**	**1**.**37**	1.41	0.80	1.80
RF	0.92	0.76	1.28	**1**.**05**	0.73	1.51	**1**.**09**	1.28	1.46	1.14	0.69	1.55
RR	1.18	0.73	1.23	1.70	0.91	1.89	1.15	1.23	1.40	1.38	0.70	1.59
Ensemble	**0**.**85**	**0**.**71**	**1**.**19**	1.33	0.80	1.66	1.09	1.24	1.41	1.39	0.77	1.73
*Middle reg.*	1.73	1.10	1.84	1.89	0.98	2.04	1.92	1.82	2.07	1.77	0.84	1.90
*Worst reg.*	4.23	2.54	4.27	4.39	2.14	4.46	4.42	3.93	4.48	4.26	1.91	4.32
*Avg. reg.*	1.47	0.98	1.65	1.86	0.97	2.02	1.55	1.48	1.69	1.81	0.86	1.94

*Middle reg.* = it always predicts 2.5, i.e., the middle score. *Worst reg.* = it always predicts the worst value (e.g., score is 1, it predicts 5, score is 3, and it predicts 0). *Avg. reg.* = it always predicts the average score among the outputs

Bold values indicates the best results

**Table 10:** Comparison of methods’ performance with respect to *Avg. Reg.* (it always predicts the average value among the outputs) on SciEntBank dataset

Method	SciEntBank			
	Improvement with respect to *Avg. reg.*			
	*q1*	*q2*	*q3*	*q4*
Ada	0.23	0.26	0.15	0.21
EN	0.00	0.03	0.17	0.24
SVR	0.17	$-$0.37	0.19	0.07
RF	0.22	0.25	0.14	0.20
RR	0.26	0.06	0.17	0.19
Ensemble	0.28	0.18	0.16	0.10

Positive numbers indicate an improvement in performance, and conversely, negative numbers indicate a worsening

## Discussion

In the following, we provide additional experiments to validate the proposed approach (Sect. 6.1). The discussion is split into two “Additional questions” paragraphs with related experiments. Next, further specifications and motivations regarding implementation choices are provided (Sects. 6.2–6.4).

### Additional experiments

*Additional question 1*

Are there significant differences between the joint analysis of lexical and semantic features and only the one of lexical features?

Table 11 shows how the answer to this question is not straightforward. We have used for each dataset, the Shapiro–Wilk [68] test to assess the normality distribution of data, and then, based on its results, we have checked the statistical difference of the performance with the two approaches with the proper test. If we accept the null hypothesis $H_0$ = “*data are normally distributed*”, the Student’s t test has been employed to check the difference [69]; otherwise, the Mann–Whitney U test [70] has been exploited (for not normally distributed data).

**Table 11:** Statistical significance of the difference in MSE results obtained with both lexical and semantic features against those got with only lexical one

Dataset	Shapiro–Wilk [68] ($\alpha =0.05$)	*t* [69], *U* [70]	*p*
ASAP	$W(120)=.93$, $p<.001$ (reject $H_0$)	$U=1317$	$p=.01132<.05$ **s**
Cu-NLP	$W(36)=.691$, $p<.001$ (reject $H_0$)	$U=98.5$	$p=.0466<.05$ **s**
SAG	$W(166)=.916$, $p<.001$ (reject $H_0$)	$U=3765$	$p=.1856>.05$ **ns**
SciEntBank	$W(44)=.95$, $p=.054$ (accept $H_0$)	$t=-0.19992$	$p=.421255>.05$ **ns**

Shapiro–Wilk test to assess normality where $H_0$ indicates that data are normally distributed. Statistical test performed: Student’s t or Mann–Whitney. Significance level is set at .05. **s** = significant at $p<.05$; **ns** = not significant at $p<.05$

**Fig. 11:**
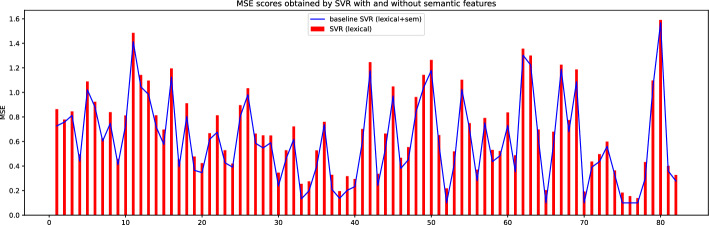
Difference of MSE obtained with and without semantic features with SVR on SAG dataset. On the x-axis, the graphic represents the questions in SAG, and on the y-axis the MSE is obtained by the SVR using different kinds of features. The blue line represents the MSE achieved with lexical and semantic features, while the red bars represent the MSE achieved with the only lexical features. Lower MSE values are better (color figure online)

**Fig. 12:**
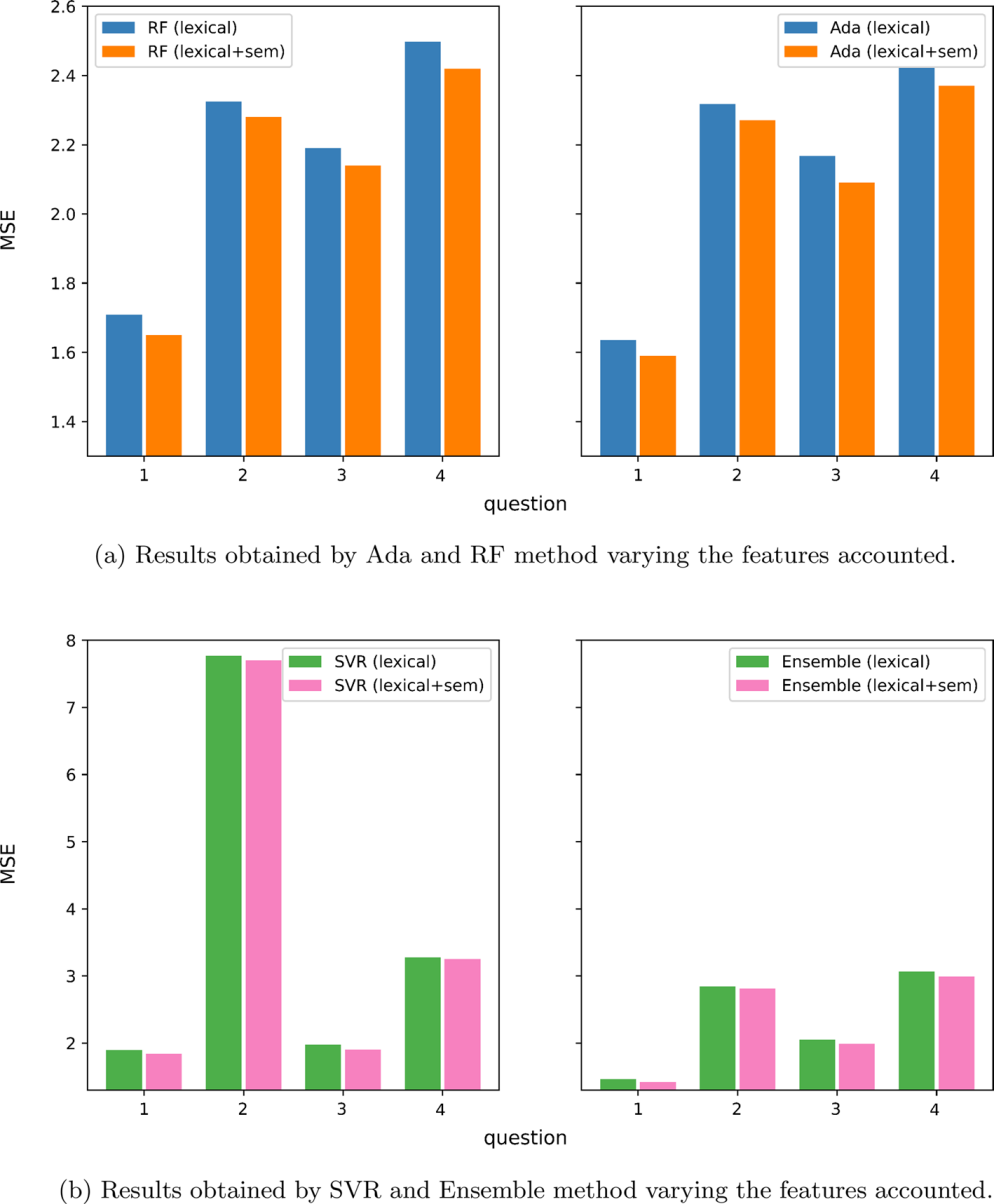
Difference of MSE obtained with and without semantic features with best regressors on SciEntBank dataset. Lower MSE values are better

These tests confirm the significance of the better results in terms of MSE, using semantic features on datasets ASAP and Cu-NLP, while the difference is not significant for SAG and SciEntBank (illustrated also in Figs. 11, 12). However, Fig. 11 shows that on the SAG dataset, although the statistical difference is not significant, performance obtained by the joint exploitation of semantic and lexical features is always better (lowering the MSE in almost every condition) than those achieved with the only lexical ones. In summary, we can consider the semantic features effective in improving the scoring precision of the methods, also if the benefits are not always high for all datasets.

*Additional question 2*

How does GradeAid perform on non-English datasets?

**Fig. 13:**
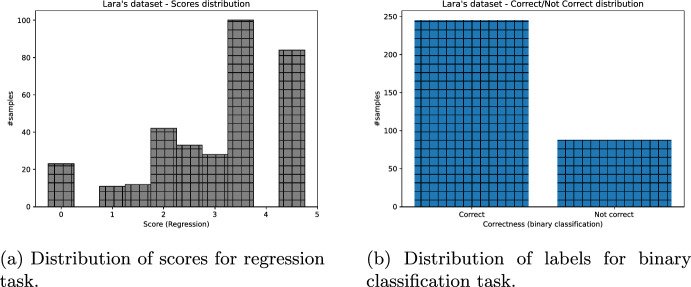
STITA’s dataset distribution of scores (and their grades counterpart)

**Fig. 14:**
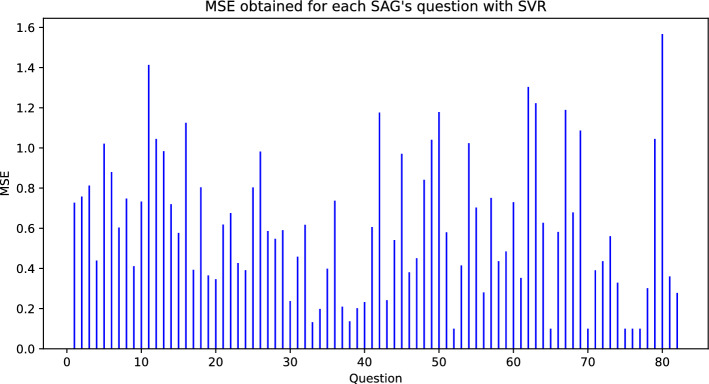
MSE achieved for each question in SAG dataset with SVR

To fully answer this research question, we have used an Italian dataset, namely STITA. The STITA dataset is made publicly available on GitHub (see Table 12). However, only the English translated version^13^ is publicly available for privacy reasons. For some preliminary information about this dataset, we refer the reader to Table 12 (where subject and number of samples are reported) and Fig. 13 (where we show the distribution of scores).

On STITA, we have performed the same experiments seen previously for other datasets (Sect. 5.1). As the results shown in Table 13, the performance of *GradeAid* is effective also on non-English datasets. The best regressor, in this case, is Ada with NRMSE from 0.54 to 0.17. This regressor shows an improvement with respect to the *Avg. reg.*, of 0.17 for *q5*, 0.21 for *q1*, 0.41 for *q2*, 0.43 for *q4* and 0.70 for *q3* (best improvement). Another very promising regressor is RF, which overcomes Ada in *q5*, showing an improvement with respect to *Avg. reg.* of 0.45.

In addition, also for STITA dataset, we have performed an experiment with and without semantic features and we have employed, as for other datasets the same statistical tests. The result, in this case, is that there is a significant difference in the results obtained using the semantic features ($p =.01511 <.05$).

These results should be take carefully, as the test has been performed only on a dataset of a specific language, i.e., Italian, translated in English with an open machine learning translator and on a specific subject, i.e., statistics. More robust proofs would involve using datasets in different languages (e.g., Arabic, German, Brazilian, etc.)—which, to the best of our knowledge, are, to date, not available for researchers, as the performance of translator could vary. Moreover, different subjects could easily impact the results. Anyway, the strategy of translating the answers and process them showed to be successful in our experimental setting.

**Table 12:** STITA dataset information

Dataset	STITA
Language	Italian
Samples	333
Subject	Statistics
Link	https://github.com/edgresearch/dataset-automaticgrading-2022

**Table 13:** Results obtained on STITA dataset with LOO-CV

Method	STITA														
	*q1*			*q2*			*q3*			*q4*			*q5*		
	MAE	NRMSE	RMSE	MAE	NRMSE	RMSE	MAE	NRMSE	RMSE	MAE	NRMSE	RMSE	MAE	NRMSE	RMSE
Ada	**0**.**46**	**0**.**19**	**0**.**71**	**0**.**49**	**0**.**17**	**0**.**64**	**0**.**28**	**0**.**17**	**0**.**42**	**0**.**58**	**0**.**23**	**0**.**93**	1.24	0.54	1.50
EN	0.54	0.21	0.75	0.99	0.40	1.44	0.62	0.30	0.73	1.08	0.32	1.26	1.18	0.53	1.46
SVR	0.53	0.21	0.76	0.79	0.25	0.91	0.40	0.25	0.61	0.90	0.30	1.18	**1**.**01**	0.49	1.36
RF	0.52	0.21	0.74	0.56	0.19	0.70	0.36	0.22	0.53	0.79	0.27	1.08	1.10	**0**.**36**	**1**.**00**
RR	0.54	0.21	0.75	0.86	0.33	1.17	0.44	0.22	0.54	0.88	0.28	1.10	1.04	0.45	1.24
Ensemble	0.49	0.20	0.72	0.70	0.24	0.86	0.38	0.25	0.61	0.85	0.28	1.10	1.08	0.49	1.36
*Middle reg.*	1.23	0.38	1.37	1.32	0.41	1.49	0.76	0.40	0.97	1.90	0.52	2.06	1.48	0.63	1.74
*Worst reg.*	3.73	1.05	3.78	3.82	1.08	3.88	3.26	1.37	3.32	4.40	1.12	4.47	3.98	1.47	4.08
*Avg. reg.*	0.73	0.24	0.87	0.81	0.29	1.05	1.12	0.56	1.34	1.42	0.40	1.58	1.45	0.65	1.80

*Middle reg.* = it always predicts 2.5, i.e., the middle score. *Worst reg.* = it always predicts the worst value (e.g., score is 1, it predicts 5, score is 3 it predicts 0). *Avg. reg.* = always predicts the average score among the outputs

Bold values indicates the best results

**Table 14:** Comparison of methods’ performance with respect to *Avg. Reg.* (it always predicts the average value among the outputs) on STITA dataset

Method	STITA				
	Improvement with respect to *Avg. reg.*				
	*q1*	*q2*	*q3*	*q4*	*q5*
Ada	0.21	0.41	0.70	0.43	0.17
EN	0.13	$-$0.38	0.46	0.20	0.18
SVR	0.13	0.14	0.55	0.25	0.25
RF	0.13	0.34	0.61	0.33	0.45
RR	0.13	$-$0.14	0.61	0.30	0.31
Ensemble	0.17	0.17	0.55	0.30	0.25

Positive numbers indicate an improvement in performance, and conversely, negative numbers indicate a worsening

### Motivation for translation in STITA experiment

A transformation/translation step somehow adds a bias in the dataset. Anyway, the machine translation used in the experiments carried, i.e., EasyNMT, is the current state of the art among the non-commercial ones. We did not use a human translation, because we wanted to test our methodology also in this way, on datasets that are not in English. Whoever intended to use an ASAG systems, it is clearly not prone to manually translate answers if they are in another language. Therefore, it is realistic that future stakeholders will use a machine translation (commercial or non-commercial), paying the price of introducing an error in the evaluation. In addition, a translation step (manual or automatic) is often required since many languages have no sophisticated embedding models available.

### Motivation for integrating lexical and semantic features

In the preliminary experiment phase we have carried out, by only using our semantic features, the performance of *GradeAid* was not promising on all datasets. Therefore, we have added lexical features that are mostly employed also in the literature of ASAG systems. Anyway, we have employed lexical features also to measure the kind of lexic is used by the student. As reported in the paper, and as also emerged from informal focus groups held with teachers and instructors from different education levels prior to the development, the exact choice of words by students is very important in several HE subjects, especially technical ones. Nonetheless, we are aware that hidden layers of BERT and other semantic models for embedding can be used to extract lexical representations. But, as highlighted by a recent EU Council’s white paper (COM(2021) 206), applications of artificial intelligence in education are considered high risk ones where the development should pursue more drillable and interpretable solutions ensuring human oversight. Therefore, since our objective was to support instructors and students, we cannot deliver a method whose outputs are hard to explain.

### Motivation for keeping the datasets’ data distribution

We kept the distribution as it is for three reasons: *(a)* Related works did not change it. To compare against them in the best possible way, we have kept the datasets distributions as it were. *(b)* After a discussion with instructors and teachers (university, high schools, etc.), we assessed that the difference in the distribution of the scores was fair for our task. *(c)* The unbalanced score of the datasets is by itself a prior of the score of answers. Of course this effect may not be wished, but for our experiments, we decided to keep it. Further experiments, and in consideration of the specific application of *GradeAid*, may induce a different choice, and using a balanced dataset may become mandatory or at least more convenient. (i.e., when the training dataset is applied in a learning environment in a different context).

## Conclusion

The presented research addresses two primary points of automatic short answer grading (ASAG) area: *(i)* a standardized pattern to benchmark the research progress and methods, and *(ii)* we proposed a new framework for ASAG, namely *GradeAid*, involving both semantic (from the BERT model) and lexical features (TF-IDF algorithm). Furthermore, differently from related works (see Sect. 2), to benchmark our performance, we experimented *GradeAid* on all the publicly available datasets in the ASAG literature. The proposed ASAG methodology mixes the traditional bag-of-words methodology with most recent progress of state-of-the-art NLP tasks. The inclusion of also semantic features, through BERT model, shows to be beneficial for all datasets accounted in this research, although in some case, the benefit has not been statistically significant.

*Expected impact*

Education and learning are the principal factors for innovation and drive wellness growth. Improvement and automation benefit the learning process and lower the costs for students education, making this more accessible also in developing countries. This research wants to be a cornerstone for ASAG by proposing a framework, a feasible standard methodology for benchmark, a new dataset and overview of the works in ASAG area. The results of this work are promising for ASAG, and the public availability of the used datasets and the code should lead to more fair and directly comparable benchmark for the proposal of new methods. In addition, it also acts as a base to be applied in production environment, overcoming the current gap in the ASAG literature.

*Limitations and future steps of the project*

Despite the promising results of *GradeAid*, the framework has some intrinsic limitations: The usage of regressors generates some paradoxes in the final scoring. Regressions are not capped, and thus, they can theoretically estimate values under the minimum (zero) and over the maximum score (five). Although a dummy solution is to manually cap the regressors’ output, this is something that would need attention in the near future. Virtually, the usage of some methods different from regressors, such as ordinal regressor, could help to address the issue and improve the quality of results: Humans do not score answers over a infinite number of scores, but across a limited set of values. Clearly, this could be addressed by rounding the output of the regressor to the nearest (integer) value. However, an ordinal regressor should be a more efficient and natural way to provide scores to students’ answers.

For the future, we aim to address these limitations by integrating our framework with other features and adopting newer datasets on specific subjects. In this research, we used both lexical and semantic features, the latter using the semantic similarity provided from a pre-trained BERT model. But such a model could be further trained with more domain-specific texts, so to improve its semantic evaluation, as performed, for example, in [34]. In addition, we are planning to experiment the latest transformer-based pre-trained models like XLM, XLM-Align, InfoXLM [71, 72]. Further directions would involve the development of a methodology able to provide comprehensibility of results following the guideline of human-centered AI [73–78]. Since the main aim of this study is to provide an automated system for feedback during learning, it is of paramount interest of learner to know what are the reasons of the score computed, to improve the quality of learning. This last point has spillovers on the cheating phenomenon [79]. On the one hand, the problem is severe, and clearly an extensive usage of automatic assessment tools like *GradeAid* could bring new challenges in facing such a problem. Currently, *GradeAid* is not intended to provide a final score for students’ answers, but as a supporting tool both for students and teachers. However, cheating is surely an aspect worth investigating for our research. To this end, before releasing *GradeAid* as a production tool we will take into consideration a series of user studies—involving students and teachers—in order to evaluate, among others, the cheating phenomenon.

For the next future, we have also planned to perform experiments with deep learning models for scoring and study advantages and disadvantages of such with respect to the current *GradeAid*.

Our ultimate goal for future is to embed *GradeAid* in a web application with an intuitive user interface. Indeed, we aim at experimenting an adaptation of *GradeAid* such that it provides contextual tags to the students’ answers. These tags are helpful for teachers and instructors to better comprehend the tool’s scores, thus enhancing the support for the final evaluation [80]. For example, an interesting evolution could be that of highlighting common keywords between students’ and reference answers.

Lastly, the hope of this paper is to nudge the research community to make more comparable, open and reproducible algorithms for ASAG.

## Data Availability

The datasets generated during and/or analyzed during the current study are available at https://github.com/edgresearch/dataset-automaticgrading-2022.

## Appendix A: Hyper-parameters tuned for each regressor

In this section, and specifically in Table 15, we show, for each regressor evaluated, the hyper-parameters tuned with the range of values and the related step. The range of the best parameters found is available in Table 16.

**Table 15:** Parameters tuned for each regressor tested

Regressor	Parameters	Range	Step
AdaBoost	*estimators*	[10, 3000]	10
	*loss*	$\{linear$, *square*, $exponential\}$	–
	$learning\_rate$	$[10^{-3},10^{3}]$	10
EN	*alpha*	[0.1, 2.0]	0.05
	$l1\_ratio$	[0, 1]	0.05
RF	$n\_estimators$	[10, 3000]	10
	*criterion*	$\{mse, mae\}$	–
RR	*solver*	$\{svd, cholesky, lsqr, sparse\_cg, sag, saga\}$	–
	*alpha*	[0.1, 2.0]	0.05
SVR	*C*	$[10^{-3},10^{3}]$	10
	*kernel*	$\{linear,$ *rbf*, $sigmoid\}$	–
	*gamma*	$[-5,+5]$	0.25

**Table 16:** Range of the best parameters found for each regressor tested on the different datasets

Regressor	Parameters	Best range
AdaBoost	*estimators*	[1110, 2100]
	*loss*	$\{linear\}$
	$$learning\_rate$$	[0.01, 1.00]
EN	*alpha*	[0.25, 1.0]
	$$l1\_ratio$$	[0.25, 0.75]
RF	$n\_estimators$	[1730, 2850]
	*criterion*	$\{mae\}$
RR	*solver*	$\{svd, sag, saga\}$
	*alpha*	[0.25, 1.0]
SVR	*C*	$[1.0,10^{2}]$
	*kernel*	$\{sigmoid, rbf\}$
	*gamma*	[0.25, 1.25]

## Appendix B: Running the experiments: pseudo-code of *GradeAid*

In this section, we sketch the (python-like) pseudo-code of *GradeAid* for running the experiments presented in the core of the paper.

First, we open the dataset provided according to the template we designed, where there must be five columns, i.e., *ID* (identifier of the question), *question* (text of the question), *reference_ans* (reference answer provided by the instructor/teacher), *student_ans* (answer provided by a student), *grade* (the evaluation provided by the teacher/instructor, from 0 to 5). Next, the dataset is split by question (line 15). For each question available, we find the indexes of samples to include in the training and validation sets (function *get_folds()* at line 19). For each folds configuration, we compute the lexical features through TF-IDF (lines 23–24) and the similarity between each students’ answer and the reference one (lines 27–28). Then, features are merged (function *fusion()* at lines 31–32). Lastly, the regressor chosen is called and after the scoring, MAE, MSE, RMSE and NRMSE are computed (lines 34–38).

**Table 17:** Results obtained on ASAP dataset with tenfold CV

Method	ASAP									
	*q1*	*q2*	*q3*	*q4*	*q5*	*q6*	*q7*	*q8*	*q9*	*q10*
	MSE									
Ada	1.53	1.66	0.57	0.74	0.65	0.80	1.34	1.34	0.75	0.75
EN	1.66	1.70	0.75	0.82	0.75	0.90	1.48	1.50	0.81	0.80
SVR	**1**.**14**	**1**.**38**	**0**.**49**	**0**.**45**	0.42	0.45	1.12	**1**.**16**	0.58	**0**.**57**
RF	1.19	1.46	0.52	0.48	**0**.**40**	**0**.**41**	**1**.**12**	1.18	**0**.**58**	0.61
RR	1.64	1.68	0.75	0.80	0.73	0.92	1.50	1.48	0.82	0.80
Ensemble	1.45	1.51	0.58	0.72	0.58	0.66	1.20	1.25	0.73	0.73
*Middle reg.*	3.00	2.71	1.85	2.70	5.08	5.51	3.56	2.25	1.92	1.60
*Worst reg.*	16.85	16.05	13.73	16.08	22.09	23.15	18.23	14.79	13.89	13.24
*Avg. reg.*	3.88	4.37	1.03	1.05	2.13	2.44	1.96	1.25	1.03	1.46

*Middle reg.* = it always predicts 2.5, i.e., the middle score. *Worst reg.* = it always predicts the worst value (e.g., score is 1, it predicts 5, score is 3, and it predicts 0)

Bold values indicates the best results

## Appendix C: Query for searching previous literature on ASAG

In the following, we report the query used to search the articles in the related work (Sect. 2).

*((automatic text based grading)* ***OR*** *(open ended questions automatic grading)* ***OR*** *(short answers automatic grading)* ***OR*** *(open ended questions automatic assessment)* ***OR*** *(short answers automatic assessment)* ***OR*** *(short answers automatic marking)* ***OR*** *(open ended questions automatic marking)* ***OR*** *(short answers automatic scoring)* ***OR*** *(open ended questions automatic scoring)* ***OR*** *(short answers machine learning grading)* ***OR*** *(natural language processing exam grading)* ***OR*** *(natural language processing exam scoring)* ***OR*** *(natural language processing exam marking)* ***OR*** *(natural language processing exam assessment)* ***OR*** *(nlp exam grading)* ***OR*** *(nlp exam scoring)* ***OR*** *(nlp exam marking)* ***OR*** *(text answers grading)* ***OR*** *(text answers marking)* ***OR*** *(essays automatic grading)* ***OR*** *(essays automatic scoring)* ***OR*** *(essays automatic marking)* ***OR*** *(essays automatic assessment)* ***OR*** *(descriptive answers automatic marking)* ***OR*** *(descriptive answers automatic grading )* ***OR*** *(descriptive answers automatic assessment))*

**Table 18:** Results obtained on Cu-NLP dataset with LOO-CV

Method	Cu-NLP	
	*q1*	*q2*
	MSE	
Ada	1.01	**0**.**67**
EN	1.19	3.60
SVR	1.18	3.00
RF	**0**.**89**	0.71
RR	1.11	3.01
Ensemble	1.03	2.06
*Middle reg.*	2.40	3.02
*Worst reg.*	14.81	17.24
*Avg. reg.*	1.21	1.78

*Middle reg.* = it always predicts 2.5, i.e., the middle score. *Worst reg.* = it always predicts the worst value (e.g., score is 1, it predicts 5, score is 3, and it predicts 0). *Avg. reg.* = it always predicts the average score among the outputs

Bold values indicates the best results

**Table 19:** Results obtained on SAG dataset with LOO-CV

Method	SAG		
	*q-worst*	*q-avg*	*q-best*
	MSE		
Ada	2.39	0.68	0.10
EN	2.40	0.74	0.20
SVR	**1**.**56**	**0**.**60**	0.10
RF	2.18	0.68	**0**.**08**
RR	2.38	0.76	0.22
Ensemble	2.27	0.70	0.13
*Middle reg.*	6.03	4.15	1.44
*Worst reg.*	24.39	19.71	12.60
*Avg. reg.*	4.67	1.20	0.26

We report here only the worst, best and the average results obtained on the questions due to the size of the dataset (83 questions). For all the results, see Fig. 14. *Middle reg.* = it always predicts 2.5, i.e., the middle score. *Worst reg.* = it always predicts the worst value (e.g., score is 1, it predicts 5, score is 3, and it predicts 0). *Avg. reg.* = it always predicts the average score among the outputs

Bold values indicates the best results

**Table 20:** Results obtained on SciEntBank dataset with LOO-CV

Method	SciEntBank			
	*q1*	*q2*	*q3*	*q4*
	MSE			
Ada	1.59	**2**.**27**	2.09	**2**.**37**
EN	2.73	3.85	1.96	2.57
SVR	1.84	7.70	**1**.**90**	3.25
RF	1.65	2.28	2.14	2.42
RR	1.53	3.57	1.96	2.51
Ensemble	**1**.**42**	2.81	1.99	2.99
*Middle reg.*	3.40	4.16	4.26	3.59
*Worst reg.*	18.27	19.85	20.10	18.66
*Avg. reg.*	2.63	4.07	2.84	3.75

*Middle reg.* = it always predicts 2.5, i.e., the middle score. *Worst reg.* = it always predicts the worst value (e.g., score is 1, it predicts 5, score is 3, and it predicts 0). *Avg. reg.* = it always predicts the average score among the outputs

Bold values indicates the best results

## Appendix D: Mean squared error obtained in the experiments

In this section, we report the MSE got for each experiment performed over the different datasets. In particular, Table 17 summarizes the MSE obtained by the different regression methods for the ASAP dataset as well as Table 18 does for Cu-NLP dataset. Likewise, Tables 19 and 20 provide the MSE achieved by the regression methods for SciEntBank and SAG datasets, respectively.

Lastly, we also provide the MSE achieved for every question in the SAG dataset by SVR method (see Fig. 14).

## Footnotes

1 https://bit.ly/3IJFuil.

2 Peter Norvig and Sebastian Thrun, “Intro to Artificial Intelligence,” https://www.udacity.com/course/intro-to-artificial-intelligence-cs271.

3 https://www.nytimes.com/2020/05/26/technology/moocs-online-learning.html.

4 https://tutor-ia.com/, https://www.gradescope.com/.

5 https://github.com/edgresearch/code-automaticgrading-2022.

6 Available at https://github.com/edgresearch/dataset-automaticgrading-2022.

7 https://www.scopus.com.

8 https://www.webofscience.com/.

9 QWK is a classification metric used when two or more raters labels (in this case, grades) are available.

10 https://scikit-learn.org.

11 https://github.com/UKPLab/EasyNMT.

12 https://github.com/nalimilan/R.TeMiS.

13 Possible limitations to the translation: the usage of autocorrection steps of the corpus answer before processing may be helpful, considering that mistypes are likely to occur when students input the answer. The dataset STITA has been corrected using the python autocorrection package before to be translated.
